# Recent studies on chemical constituents of *Ophiorrhiza* plants

**DOI:** 10.1007/s11418-022-01640-3

**Published:** 2022-07-28

**Authors:** Mariko Kitajima

**Affiliations:** Graduate School of Pharmaceutical Sciences, Chiba University, 1-8-1 Inohana, Chuo-ku, Chiba, 260-8675 Japan

**Keywords:** Alkaloid, *Ophiorrhiza*, Rubiaceae, Camptothecin, Biosynthesis

## Abstract

*Ophiorrhiza* plants (Family Rubiaceae) are known to produce diverse monoterpenoid indole alkaloids including camptothecin with potent antitumor activity. This review contains a summary of recent chemical studies reported over the past 10 years regarding alkaloids (monoterpenoid indole and tetrahydroisoquinoline alkaloids, and cyclopeptide) in *Ophiorrhiza* plants. In addition, the alkaloid biosynthetic pathways based on their reported structures were proposed.

## Introduction

*Ophiorrhiza* plants belong to the Rubiaceae family and are widely distributed in tropical and subtropical Asia, Australia, New Guinea, and the Pacific Islands. Some of *Ophiorrhiza* plants have been used traditionally to treat snakebites, ulcers, skin disorders, etc. [1, 2]. *Ophiorrhiza* species are known to produce diverse monoterpenoid indole alkaloids including camptothecin with potent antitumor activity [1–5]. Camptothecin biosynthesis has been investigated at the genetic level, and its biotechnological production continues to attract research interest [6–10]. This review contains a summary of recent chemical studies on alkaloids isolated from *Ophiorrhiza* plants over the past 10 years. In addition, the biosynthetic pathways for some isolated monoterpenoid indole and tetrahydroisoquinoline alkaloids based on their reported structures were proposed.

## Monoterpenoid indole alkaloid glycosides from *Ophiorrhiza trichocarpon*

**Fig. 1:**
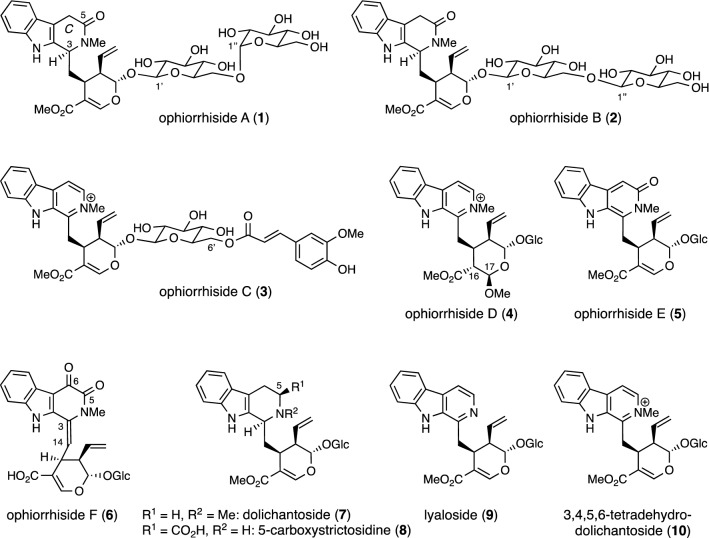
Structures of ophiorrhisides A–F (**1**–**6**) and alkaloids **7**–**10** isolated from *Ophiorrhiza trichocarpon*

In 2013, ophiorrhisides A–F (**1**–**6**), β-carboline-type monoterpenoid indole alkaloid glycosides, were isolated from *Ophiorrhiza trichocarpon* collected in Thailand, together with four known alkaloid glycosides [dolichantoside (**7**), 5-carboxystrictosidine (**8**), lyaloside (**9**), and 3,4,5,6-tetradehydrodolichantoside (**10**)] (Fig. 1) [11]. Ophiorrhisides A (**1**) and B (**2**) both possess a lactam moiety on the *C* ring and a disaccharide residue. The stereochemistry at C-3 of **1** was concluded to be *S* form, deduced from biogenetic considerations and comparison of its electronic circular dichroism (ECD) spectrum with that of a chiral model compound possessing a 1,2,3,4-tetrahydro-β-carbolin-3-one skeleton [12]. Ophiorrhiside C (**3**) with an *E*-ferulate residue on the sugar portion and ophiorrhiside D (**4**) with a fully substituted tetrahydropyran ring are analogs of 3,4,5,6-tetradehydrodolichantoside (**10**), which was also isolated from the same plant. Ophiorrhisides E (**5**) and F (**6**) each have an impressive *C* ring. Thus, the former has an *N*-methylpyridone ring. The latter has a highly oxidized *C* ring with a 1,2-dicarbonyl function at C-5 and C-6, and a double bond belonging to enamine between C-3 and C-14.

## A cyclopeptide, monoterpenoid indole alkaloid glycosides, and monoterpenoid tetrahydroisoquinoline alkaloids from *Ophiorrhiza nutans*

**Fig. 2:**
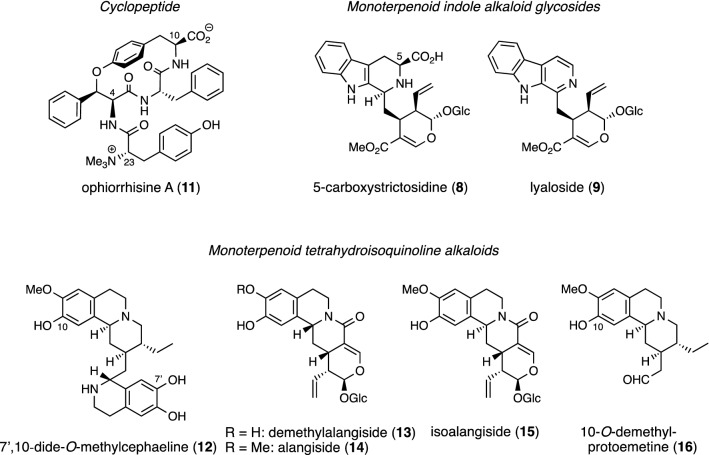
Structures of ophiorrhisine A (**11**), 7’,10-dide-*O*-methylcephaeline (**12**), and alkaloids **8**, **9**, and **13**–**16** isolated from *Ophiorrhiza nutans*

In 2017, ophiorrhisine A (**11**), a cyclopeptide, and 7’,10-dide-*O*-methylcephaeline (**12**), a monoterpenoid tetrahydroisoquinoline alkaloid, were isolated from *Ophiorrhiza nutans* collected in Thailand, together with two known monoterpenoid indole alkaloid glycosides [5-carboxystrictosidine (**8**) and lyaloside (**9**)] and four known tetrahydroisoquinoline alkaloids [demethylalangiside (**13**), alangiside (**14**), isoalangiside (**15**), and 10-*O*-demethylprotoemetine (**16**)] (Fig. 2) [13]. Among these, 5-carboxystrictosidine (**8**), demethylalangiside (**13**), and alangiside (**14**) were isolated as main alkaloids. This is the first example of monoterpenoid tetrahydroisoquinoline alkaloids isolated from the genus *Ophiorrhiza*, and the second example of monoterpenoid indole alkaloids co-existing with monoterpenoid tetrahydroisoquinoline alkaloids in the same plant species.

**Fig. 3:**
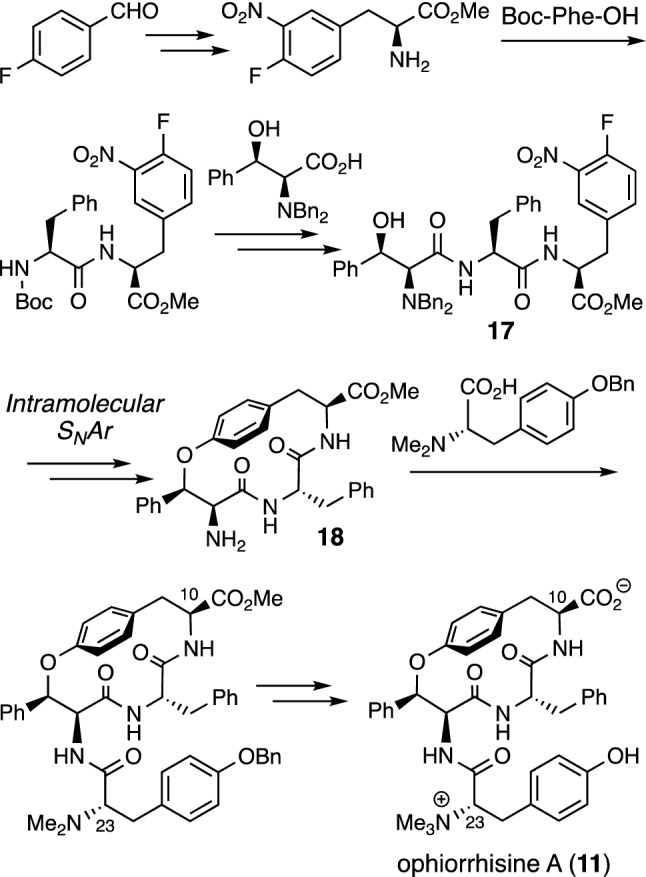
Asymmetric total synthesis of ophiorrhisine A (**11**)

**Fig. 4:**
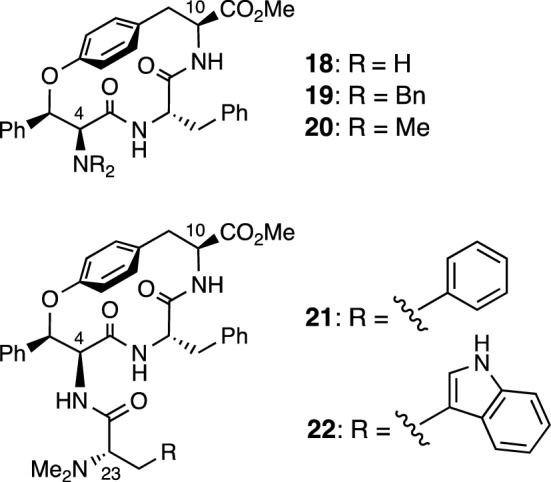
Structures of ophiorrhisine A derivatives having cytotoxicity against human cancer cell lines

Ophiorrhisine A (**11**) is a cyclic tetrapeptide having a 14-membered paracyclophane ring. The characteristic functionalities of **11** include a carboxylate group at C-10 and an *N*,*N*,*N*-trimethylated tyrosine residue in the side chain. The structure and absolute configuration of **11** were determined by spectroscopic analyses and asymmetric total synthesis involving an intramolecular aromatic nucleophilic substitution reaction (S_N_Ar) of linear tripeptide **17** to construct a 14-membered paracyclophane ring (Fig. 3) [14]. No cytotoxic activity was observed for naturally occurring **11** with an ionic character against cancer cells. On the other hand, some 14-membered cyclophane derivatives without ionic character, such as primary amine derivative **18**, *N*,*N*-dibenzyl derivative **19**, *N*,*N*-dimethyl derivative **20**, phenylalanine derivative **21**, and tryptophan derivative **22**, showed cytotoxicity against various types of human cancer cell lines (A549, HT29, HCT116) with IC_50_ values in the range of 2.9–11.6 µM (Fig. 4).

## Monoterpenoid indole alkaloid glycosides from *Ophiorrhiza japonica*

**Fig. 5:**
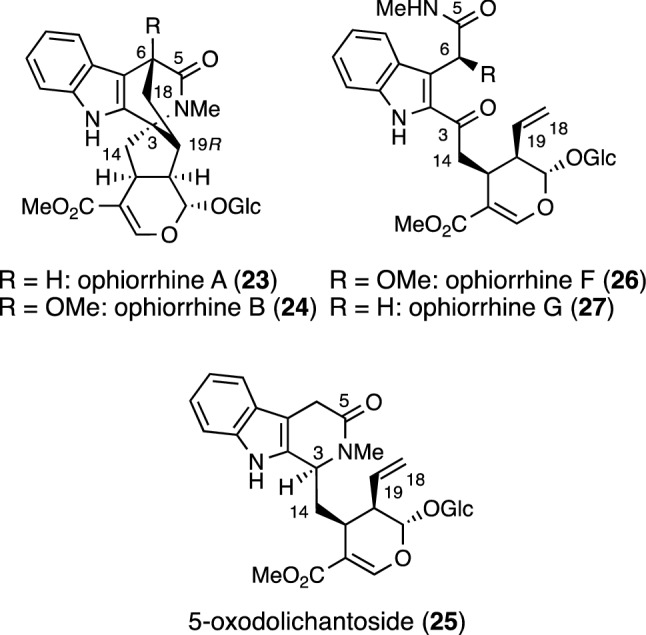
Structures of ophiorrhines A (**23**), B (**24**), F (**26**), and G (**27**) and alkaloid **25** isolated from *Ophiorrhiza japonica*

The isolation of several alkaloids from *Ophiorrhiza japonica* has been reported. In 2018, ophiorrhines A (**23**) and B (**24**), immunosuppressive monoterpenoid indole alkaloid glycosides, were isolated from *O. japonica*, a folk herbal medicine collected in China (Fig. 5) [15]. The structures of **23** and **24** were elucidated by spectroscopic analyses and single-crystal X-ray diffraction. Both **23** and **24** possess a novel spirocyclic ring system and bridged carbon ring system. The major alkaloid isolated from the plant is 5-oxodolichantoside (**25**). The same group isolated ophiorrhines F (**26**) and G (**27**) from *O. japonica* in 2022, and proposed a biosynthetic pathway for ophiorrhines A (**23**) and B (**24**) via **26** and **27** as described (vide infra) [16]. The structures of **26** and **27** were elucidated by spectroscopic methods, ECD, and calculated NMR with DP4 + analysis. Then, the inhibitory activity against lipopolysaccharide (LPS)-induced B cell proliferation was observed with ophiorrhines A (**23**), B (**24**), F (**26**), and G (**27**); the IC_50_ value of **26** was 0.38 μM. Furthermore, ophiorrhine B (**24**) also showed potent inhibitory activity against concanavalin A (Con A)-induced T cell proliferation, with an IC_50_ value of 13.34 μM. In addition, compounds **23** and **24** showed no cytotoxic activity against five human cancer cell lines HL-60, A549, SMMC-7721, SW480, and MCF-7.

## Alkaloids from *Ophiorrhiza cantoniensis*

**Fig. 6:**
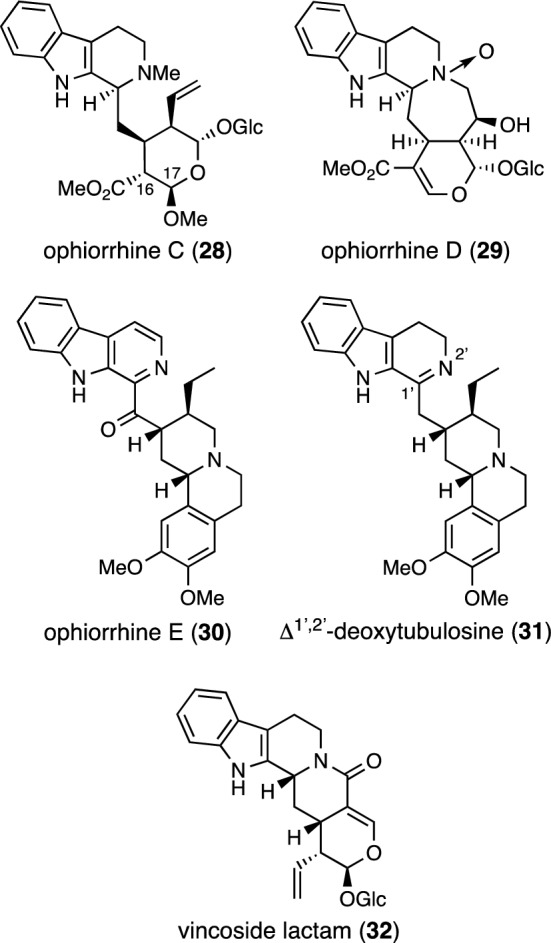
Structures of ophiorrhines C–E (**28**–**30**) and alkaloids **31** and **32** isolated from *Ophiorrhiza cantoniensis*

In 2021, ophiorrhines C–E (**28**–**30**) were isolated from *Ophiorrhiza cantoniensis* collected in China, together with one known alkaloid Δ^1’,2’^-deoxytubulosine (**31**) (Fig. 6) [17]. The structures of **28**–**30** and their absolute configurations were elucidated by spectroscopic methods, ECD, and calculated NMR with DP4 + analysis. The relative configuration of **28** at C-16 was assigned by gauge-independent atomic orbital (GIAO) ^13^C NMR calculations and DP4 + analysis. Immunosuppressive activity assays demonstrated the inhibitory activity of compounds **28** and **29** against Con A-induced T cell proliferation with IC_50_ values of 23.6 and 17.9 μM, respectively, and the inhibitory activity of **28** against LPS-induced B cell proliferation with an IC_50_ value of 8.7 μM. Vincoside lactam (**32**) was also isolated from *O. cantoniensis* [18].

## Alkaloids from other *Ophiorrhiza* plants

**Fig. 7:**
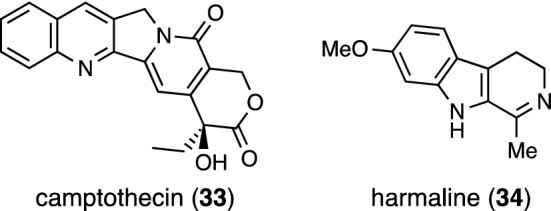
Structures of camptothecin (**33**) and harmaline (**34**)

Camptothecin (**33**) is a well-known alkaloid with potent antitumor activity (Fig. 7). It was first isolated from *Camptotheca acuminata* (Nyssaceae) in 1966 and after that, isolated from several *Ophiorrhiza* plants. In 2016, **33** was isolated from *O. shendurunii* collected in South India [19]. Compound **33** was also detected in *O. mungos* var. *angustifolia* collected in India [20], field-grown plants of *O. pectinata* [21], and *O. cantoniensis* cultivated by hydroponics [22]. Searches for camptothecin-producing *Ophiorrhiza* species in India using quantification of **33** using HPTLC-densitometry have been reported [23, 24].

Vincoside lactam (vincosamide) (**32**) and 5-carboxystrictosidine (**8**) were isolated from *O. baviensis* collected in Vietnam [25]; the inhibitory effect on NO production in LPS-stimulated RAW264.7 cells of **8** was found. Harmaline (**34**) was isolated from *O. nicobarica*, a traditional herb collected in India, and was shown to have anti-herpes simplex virus type 2 (HSV-2) activity [26] and anti-HSV-1 activity [27] in biological evaluations.

## Plausible biosynthetic pathways for monoterpenoid indole and tetrahydroisoquinoline alkaloids in *Ophiorrhiza* plants

**Fig. 8:**
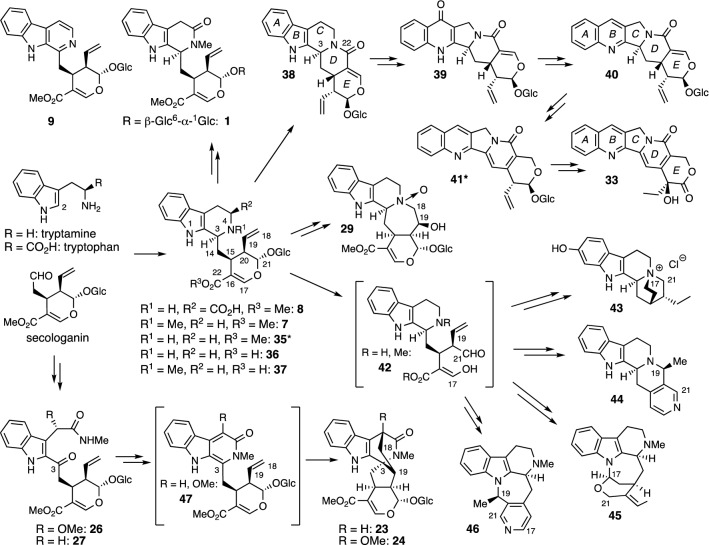
Plausible biosynthetic pathways of some monoterpenoid indole alkaloids in *Ophiorrhiza* plants (* not isolated from *Ophiorrhiza* plants but found in other plants)

Roughly 50 alkaloids have been isolated from various *Ophiorrhiza* plants. Plausible biosynthetic pathways for some of the isolated monoterpenoid indole alkaloids based on their reported structures are summarized in Fig. 8 [28]. The condensation of tryptamine with secologanin produces strictosidine (**35**), a common intermediate of monoterpenoid indole alkaloids. Strictosidine **35** itself has never been isolated from *Ophiorrhiza* plants, but strictosidinic acid (**36**), a carboxylic acid congener, has been isolated from *O. filistipula* [29]. Thus, compound **35** is utilized in this biosynthetic pathway. 5-Carboxystrictosidine (**8**) having a carboxyl group at C-5 would be formed from the reaction of secologanin and tryptophan instead of tryptamine. Alkaloids such as ophiorrhisides A–F (**1**–**6**), lyaloside (**9**), 3,4,5,6-tetradehydrodolichantoside (**10**), and ophiorrhine C (**28**) would be derived from strictosidine (**35**) or its congeners dolichantoside (**7**), **36**, and palicoside (**37**) [30, 31] without additional ring formation. Lactam formation between N-4 and the methyl ester group of strictosidine (**35**) is considered to give strictosamide (**38**) [32, 33]. Pumiloside (**39**) [32–34] and 3*S*-deoxypumiloside (**40**) [35] possess both the 6–6-5 (*ABC*)-ring system like camptothecin (**33**) and the *DE*-ring moiety as strictosamide (**38**). In 2015, camptothecoside (**41**), which has the same *ABCD*-ring system as **33** and the *E* ring acetal glucoside moiety as **38**, was isolated from *Camptotheca acuminata* [36]. Thus, camptothecin (**33**) would be derived from strictosamide (**38**) via the formation of pumiloside (**39**), 3*S*-deoxypumiloside (**40**), and camptothecoside (**41**) followed by structural conversion of the *E* ring [5]. Ophiorrhine D (**29**) with a seven-membered azepane ring would be formed from **35** via epoxidation of the C-18–C-19 double bond followed by nucleophilic addition of N-4 to C-18. Cleavage of the glucose unit in **35**, **7**, or **37** would give aldehyde intermediate **42**, from which alkaloids **43**–**46** and related compounds might be produced. (1) Ophiorrhizine (**43**) [37] would be formed via cyclization of N-4 and both carbons at C-21 and C-17. (2) Normalindine (**44**) [29] would be derived by bond formation between N-4 and C-19 and incorporation of the third nitrogen atom. (3) Dihydrocycloakagerine (**45**) [38] would be produced via cyclization of N-1 and C-17 and formation of an ether linkage between C-17 and C-21 to form a hemiaminal ether moiety. (4) 3,14-Dihydrodecussine (mostueine) (**46**) [38] would be derived by bond formation between N-1 and C-19 and incorporation of the third nitrogen atom. The biosynthetic pathway for ophiorrhines A (**23**) and B (**24**) via ophiorrhines F (**26**) and G (**27)** was proposed by Feng and Liu et al. in 2021 [16]. Thus, the condensation at the C-2 position of tryptamine and secologanin (or secoxyloganin derivative) would yield ophiorrhines F (**26**) and G (**27**), respectively, which would then be metabolized into ophiorrhines A (**23**) and B (**24**), respectively, via an intramolecular [4 + 2] Diels–Alder cycloaddition of hypothetical intermediate **47**.

**Fig. 9:**
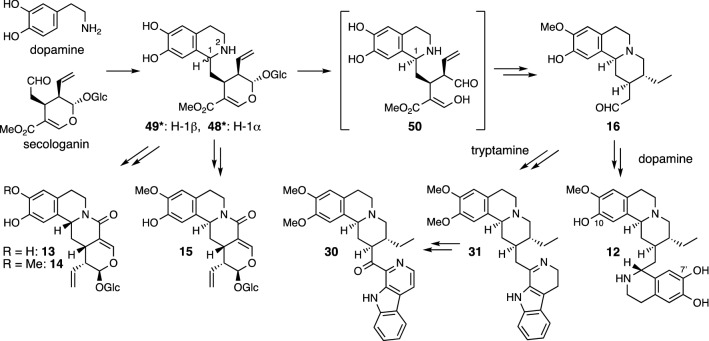
Proposed biosynthetic pathways for monoterpenoid tetrahydroisoquinoline alkaloids in *Ophiorrhiza* plants (* not isolated from *Ophiorrhiza* plants but found in other plants)

The proposed biosynthetic pathways for monoterpenoid tetrahydroisoquinoline alkaloids in *Ophiorrhiza* plants are shown in Fig. 9. The condensation of dopamine with secologanin is considered to produce deacetylisoipecoside (**48**) with H-1α and deacetylipecoside (**49**) with H-1β, although neither **48** nor **49** has been isolated from *Ophiorrhiza* plants to date. Isoalangiside (**15**) having H-1α and demethylalangiside (**13**) and alangiside (**14**) having H-1β would be formed from **48** and **49**, respectively, via lactam formation between N-2 and the methyl ester group. On the other hand, the hydrolysis of the glucose unit in **48** and piperidine ring formation in the resulting aldehyde intermediate **50**, followed by a sequence of reactions, would give 10-*O*-demethylprotoemetine (**16**). The reaction of **16** or its analogs with a second dopamine would yield 7’,10-dide-*O*-methylcephaeline (**12**), whereas the reaction of **16** with tryptamine would lead to the formation of ophiorrhine E (**30**) via Δ^1’,2’^-deoxytubulosine (**31**).

## Conclusion

This review contains a summary of chemical studies reported over the past 10 years regarding the alkaloidal constituents of *Ophiorrhiza* plants. A number of alkaloids having unique chemical structures have been isolated, including monoterpenoid indole alkaloid glycosides, monoterpenoid tetrahydroisoquinoline alkaloids, and a cyclopeptide. Among them, some (including synthetic analogs) have demonstrated useful biological activities. The second half of this review discussed the plausible biosynthetic pathways for the isolated monoterpenoid indole and tetrahydroisoquinoline alkaloids based on their reported structures. Their diverse chemical structures would be derived from common intermediates obtained by the condensation of secologanin with tryptamine (tryptophan) or dopamine. It is highly anticipated that the candidate biosynthetic intermediates of the related alkaloids and novel alkaloids having unique skeletons and biological activities would be discovered from *Ophiorrhiza* plants in the future.
